# Prevalence of depressive symptoms and associated factors among older adults living in aged care homes of Kathmandu Metropolitan City, Nepal

**DOI:** 10.1371/journal.pgph.0003359

**Published:** 2024-11-25

**Authors:** Sanju Maharjan, Nujan Tiwari, Sita Bista, Prem Basel

**Affiliations:** 1 Central Department of Public Health, Institute of Medicine, Tribhuvan University, Kathmandu, Nepal; 2 Department of Community Medicine and Public Health, Maharajgunj Medical Campus, Institute of Medicine, Tribhuvan University, Kathmandu, Nepal; African Population and Health Research Center, KENYA

## Abstract

Depressive symptoms have become a global public health problem, with a predominant effect on the older adults. The studies on mental health status of older adults in Nepal are quite limited. In this study, we aim to assess the prevalence of depressive symptoms and associated factors among the older adults living in public aged care homes of Kathmandu Metropolitan City in Nepal. A cross-sectional study was conducted among 142 older adults; aged 60 years and above recruited through proportional simple random sampling from six aged care homes. The 15 item-Geriatric Depression Scale (GDS) was used to assess the depressive symptoms among the participants. Associated factors were tested using Chi-square test; and a p-value of less than 0.05 with a confidence interval of 95% was used for statistical significance. More than half of the study participants, 58.5% (95% CI: 49.9%-66.7%) were found to have depressive symptoms. Among them, 38.7% had mild symptoms, 16.2% had moderate symptoms and 3.5% had severe symptoms. Age (OR = 2.25, 95% CI: 1.08–4.66), sex (OR = 2.36, 95% CI: 1.17–4.75), past family type (OR = 0.44, 0.22–0.89), chronic physical health problem (OR = 0.34, 95% CI: 0.12–0.98) and feelings of loneliness were found to have significant association with depressive symptoms among the older adults’ population. The prevalence of depressive symptoms among the older adults in aged care homes in Kathmandu Metropolitan City is quite high and is found to be associated with age, sex, past family type, chronic physical health problems and feeling of loneliness. It is a concerning issue that requires targeted mental health programs and interventions in order to bring about a positive shift in their mental health condition. It also demands a robust collaboration between the local bodies, health institutions, administrators, private as well as nonprofit institutions to bring desirable change.

## Introduction

The World Health Organization (WHO) defines depression as a mental health disorder that presents with persistently depressed mood, loss of interest or pleasure in previously pleasurable activities, decreased energy, feelings of guilt or low self-worth, disturbed sleep or appetite, and poor concentration [1]. While there is a difference between depression and depressive symptoms, depressive symptoms could be crucial in diagnosing depression and address mental health issues. Depressive symptoms can affect the quality of life and is one of the leading causes of disability worldwide [2, 3]. The older adults are at an even higher risk of mental health problems including anxiety and depression, with depression being the most common psychiatric disorder in later life [3, 4]. A meta-analysis of studies from around the world revealed the prevalence of depression to be 31.74%, with prevalence higher in developing countries (40.78%) than in the developed countries (17.05%) [5].

Deep rooted social stigma, misconceptions and serious inadequacies with regards to information, resources, health facilities, all act as barriers to accessing proper mental health services, especially in developing countries. The Government of Nepal has a limited number of trained human resources and funds for the effective and efficient implementation of the national policy, act and regulations on ageing and the problems of the older adults. As such, the unique health needs of older adults have largely been neglected [6]. Further, a 3.5% population growth rate of the older adults is a challenge to the existing healthcare demand in the country [7].

While it is true that additional years of life open up a wide range of opportunities and contributions to society, we cannot be oblivious to the fact that it all depends on the health of the population [8]. Multiple risk factors leading to mental health problems at any point in life have been identified but the stressors that have been deemed significant among older adults include depreciation of physical, social and cognitive functions from illness, retirement, bereavement, loss of income and disability [8, 9]. The presence of one or more of these risk factors often result in isolation, loneliness and distress in older adults that require a significant amount of care [3, 8]. They are commonly perceived as a burden to family and society, making them all the more vulnerable to physical and emotional abuse, abandonment and financial crisis. This compels them to opt for other places to live a free and respectful life, and aged care homes are often their only support to do so [4, 10]. Depressive symptoms have been found to be more common in institutionalized older adults. As these symptoms usually coexist with other health problems, mental health symptoms are overlooked and often left untreated [3, 9].

There have been a few studies conducted in Nepal among the older adults. Senior Citizens Act, 2006 in Nepal regards individuals aged 60 or above as senior citizens or older adults. In a study conducted by Chalise, the prevalence of depression among older adults living in old age homes of Devghat area of Nepal was found to be 57.8% [11]. A study by Ghimire et al comparing the prevalence of depression between older adults living in old age homes and those living in the community reported 52.73% prevalence in the old age homes which was twice of that in the community; 25.45% [12]. Another study by Dhungana et al found presence of depression among 80.7% of the older adults participating in their study [13]. All the previous studies suggested that age, gender, ethnicity, poor perception of life, poor social relationships, chronic organic diseases, lack of entertainment activities were associated with higher levels of depression. Kathmandu Metropolitan City houses the largest and most diverse population demographics in the country and also has the greatest number of operational aged care homes in Nepal. There is limited data regarding the prevalence of depression in these institutions. Thus, this study aims to assess the prevalence of depressive symptoms (outcome) and its associated factors among the older adults (predictor) living at aged care homes in Kathmandu Metropolitan City, Nepal.

## Methodology

### Study design and participants

This is an institutional based cross-sectional study conducted in six aged care homes in Kathmandu Metropolitan City. After receiving ethical approval from the Institutional Review Board (IRB) of IOM on 16 March 2022 [Approval number: 371 (6–11) E^2^ 078/079], participant recruitment and data collection was conducted from 22 March 2022 to 5 April 2022.

### Inclusion and exclusion criteria

The study population consists of the adults of age 60 years and over, of all public aged care homes in the study site. The older adults who could not communicate verbally and who did not understand Nepali language were excluded from the study. The participants who did not consent for the study and were in ill health were also excluded.

### Sample size

The total calculated sample was 142 with assumptions {Population in 6 aged care homes in Kathmandu Metropolitan City = 198, permissible error = 0.05, Standard normal variate = 1.96 at 95% confidence interval, prevalence = 0.578 taken from the study by Chalise [11], non-response rate = 10%}. The proportion of the sample from the population size was 71.72%.

### Sampling procedure

**Fig 1:**
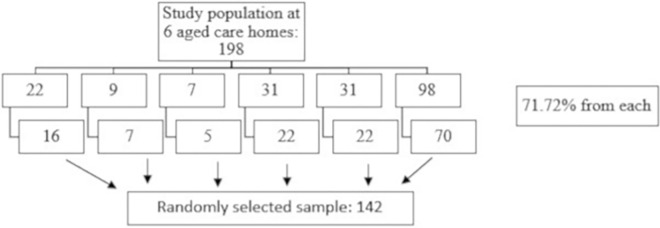
Sampling process for the study.

There are 6 aged care homes working in coordination with Kathmandu Metropolitan City. Proportionate simple random sampling was applied to select the participants. The proportion of sample from the total population is 71.72%. There are 22, 9, 7, 31, 31, 98 older adults living in 6 aged care homes of Kathmandu Metropolitan City. So, the sample proportion for the study in each aged care home was 16, 7, 5, 22, 22, and 70 respectively. The sampling process is presented in Fig 1.

### Study measures

#### Dependent variable

- Depression

#### Independent variables

- Socio-demographic characters of the older adults: Age, sex, ethnicity, marital status, education, past family type, present source of income
- Health status: Presence of chronic medical illnesses
- Individual factors: Feelings of loneliness

Data collection was done with face-to-face interview with selected participants from the representative sample. The tool was pretested in one of the aged care homes outside the study area. Structured and validated short form of Geriatric Depression Scale (GDS 15) was used to assess depressive symptoms [14]. It consists of a 15 ‘Yes/No’ questions to measure depressive symptoms. Scoring range of 0 to 4 is considered to be normal, 5 to 8 is mild, 9 to 11 is moderate and 12 to 15 indicates severe depression [15].

### Statistical analysis

Collected data was entered in Epidata version 3.1 software and exported to SPSS v20 for further analysis. The questions were scored accordingly to calculate the total score and level of depressive symptoms. For further analysis, the variables were dichotomized into normal and presence of depressive symptoms. The association between depressive symptoms and different variables was tested using the chi-square test. If p-value was less than 0.05 at a 95% confidence interval, then the association between dependent and independent variables was considered statistically significant.

### Ethical consideration

Ethical approval was obtained from the Institutional Review Committee (IRC) of the Institute of Medicine (IOM). Official letter of cooperation from the Central Department of Public Health was written to the administrative office of the Kathmandu Metropolitan City and permission was also obtained from each of the aged care homes regarding data collection from their residents. Verbal informed consent was obtained from all study participants as it had been informed by the administrators that all older adults could not read and write and obtaining thumb print was not a feasible task.

## Results

A total of 142 older adults participated in the study. The mean age of the participants was 76.37 years (SD: 8.21 years). Majority of the participants of our study were females (64.1%), with 35.9% being males. 52.8% of the participants were of Brahmin/Chhetri ethnicity while the rest were Janajatis (indigenous groups). A majority of the older adults (61.3%) had already lost their partners during the study. More than half, 56.3% lived with joint family in the past and 57% had at least one source of income. 90.1% of the participants could not read and write.

With regards to their physical health; the majority, 63.2% had musculoskeletal problems, followed by hypertension (26.4%), gastro-enteric problems (21.6%), diabetes mellitus (21.6%), respiratory problems (20.8%) and others (4%). The responses regarding physical health were based on the individual responses as well as consultation with nurses in the aged care homes for verification. Table 1 summarizes the socio-demographic characteristics of the respondents.

**Table 1:** Socio-demographic characteristics of the respondents.

Characteristics	Number	Percentage
**Age in years** (Mean (± SD) = 76.37±8.21)		
60–79	91	64.1
80 and above	51	35.9
**Sex**		
Male	51	35.9
Female	91	64.1
**Ethnicity**		
Janajati	67	47.2
Brahmin/Chhetri	75	52.8
**Marital status**		
Single	22	15.5
Married	17	12.0
Widow/widower	87	61.3
Separated/divorced	16	11.3
**Education status**		
Can read and write	14	9.9
Cannot read and write	128	90.1
**Past family type**		
Nuclear	62	43.7
Joint	80	56.3
**Source of income**		
Yes	81	57.0
No	61	43.0
**Type of source** *		
Pension	1	1.1
Older adults’ allowance	72	79.1
Savings	3	3.3
Business	15	16.5

*Multiple response

n = 142

### Health status of the respondents

Table 2 shows that a large group, 88% of the respondents had chronic health problems. Musculoskeletal problems were the most prominent one followed by hypertension, gastroenteric and diabetes, respiratory problems and others (which included skin conditions and congenital heart problems).

**Table 2:** Health status of the respondents.

Characteristics	Number	Percentage
**Chronic health problems**		
Yes	125	88
No	17	12
**Type of Problem** *		
Gastroenteric problems	27	21.6
Respiratory problems	26	20.8
Hypertension	33	26.4
Diabetes	27	21.6
Musculoskeletal problems	79	63.2
Others	5	4

*Multiple response

n = 142

### Individual factor of the respondents

Feeling of loneliness was studied under individual factor. The results in Table 3 shows 45.5% of the respondents shared that they felt lonely.

**Table 3:** Individual factor of the respondents.

Characteristics	Number	Percentage
**Feeling of loneliness**		
Yes	65	45.8
No	77	54.2

n = 142

### Prevalence of depressive symptoms among older adults living in aged care homes

As detailed in Table 4, the study revealed overall prevalence was found to be 58.5% (95% CI: 49.9%-66.7%). 38.7% older adults were found to have mild depressive symptoms, followed by 16.2% with moderate symptoms and 3.5% with severe depressive symptoms.

**Table 4:** Prevalence of depressive symptoms among older adults.

Level of depressive symptoms	Number	Percentage
Normal	59	41.5
Mild	55	38.7
Moderate	23	16.2
Severe	5	3.5

n = 142

### Association of risk factors with depressive symptoms

Table 5 represents the association of depressive symptoms with different variables. It was found that age, sex and past family type were statistically significant with depressive symptoms. Older adults of age 80 and above were 2.25 times more likely to have depressive symptoms than those of age 60–79 (OR = 2.25, 95% CI: 1.08–4.66). Similarly, females were 2.36 times more likely to have symptoms than males (OR = 2.36, 95%CI: 1.17–4.75). Likewise, older adults who previously lived with joint families were found to be protected against depressive symptoms in comparison to those living in nuclear families (OR = 0.44, 0.22–0.89).

**Table 5:** Association between depressive symptoms and risk factors.

Characteristics	Normal n (%)	Depressive Symptoms n (%)	χ^2^ value	p value	OR (95% CI)
**Age**					
60–79 years	44(48.4)	47(51.6)	4.828	**0.028** *	2.25
80 and above	15(29.4)	36(70.6)			(1.08–4.66)
**Sex**					
Male	28(54.9)	23(45.1)	5.843	**0.016** *	2.36
Female	31(34.1)	60(65.9)			(1.17–4.75)
**Ethnicity**					
Janajati	32(47.8)	35(52.2)	2.016	0.156	1.63
Brahmin Chhetri	27(36)	48(64)			(0.83–3.18)
**Marital status**					
Single	8(36.4)	14(63.6)	1.328	0.722	
Married	7(41.2)	10(58.8)			
Widow/widower	39(44.8)	48(55.2)			
Separated/divorced	5(31.3)	11(68.8)			
**Education status**					
Can read and write	5(35.7)	9(64.3)	0.218	0.641	0.76
Cannot read and write	54(42.2)	74(57.8)			(0.24–2.40)
**Past family type**					
Nuclear	19(30.6)	43(69.4)	5.388	**0.020** *	0.44
Joint	40(50)	40(50)			(0.22–0.89)
**Source of income**					
Yes	35(43.2)	46(56.8)	0.214	0.644	1.17
No	24(39.3)	37(60.7)			(0.60–2.31)
**Suffering from chronic problem**					
Yes	48(38.4)	77(61.6)	4.624	**0.039** *	0.34
No	11(64.7)	6(35.3)			(0.12–0.98)
**Feeling of loneliness**					
Yes	19(29.2)	46(70.8)	7.490	**0.006** *	0.38
No	40(51.9)	37(48.1)			(0.19–0.77)

*Statistically significant (p<0.05)

n = 142

Suffering from a chronic physical health problem (p = 0.039) was found to be statistically significant for depressive symptoms. Older adults who suffered from chronic physical health problems were found to be more prone to have depressive symptoms (OR = 0.34, 95% CI: 0.12–0.98). The feeling of loneliness (p = 0.006) was found to be statistically significant with depressive symptoms. Having feelings of loneliness made them vulnerable to depressive symptoms (OR = 0.38, 95% CI: 0.19–0.77).

## Discussion

Depression is an important public health problem but the stigma and ignorance lingering around it prevents the true statistics of depression from being studied. Early identification and diagnosis of depressive symptoms help improve quality of life of the sufferer. Through this study, we aimed to determine the prevalence of depressive symptoms among the older adults living in aged care homes of Kathmandu Metropolitan City and ascertain the association between depressive symptoms and study characteristics. Our study shows association of depressive symptoms with age, sex, past family type, chronic physical health problems and feelings of loneliness.

58.5% of the respondents in our study were found to have depressive symptoms. Regarding its severity, mild depression was the most common (38.7%), followed by moderate symptoms (16.2%) while 3.5% of the older adults had severe symptoms. Another study conducted among the older adults in old age homes of Kathmandu valley by Kafle et al had found the prevalence of depression to be 47.3%, of which 34% had mild depression and 13.3% had severe depression [16]. These variations could be due to variation in the study settings and coverage of a larger study area. Another study looking at the association of depression with the quality of life among the older adults had revealed the prevalence of depression to be 39.6% [17]. Increased prevalence rate in our study could reflect the impacts of COVID-19 pandemic which led to mobility restrictions imposed in the aged care homes. This seems to be slightly higher than the rates of depression in India and Bangladesh [18, 19].

Our study reports a higher prevalence of depressive symptoms among the females. This finding is consistent with other studies in this realm [11, 17]. Increasing age was considered an important predictor of increasing depression in a study conducted by Zou et al among the inpatients of a tertiary care center in China [20]. Being a female had greater association with depressive symptoms as suggested by other studies too [11, 13, 21]. This might also be a result of lesser opportunities to females and them being more susceptible to discrimination in our society, which leads to a lower self-esteem and generates a feeling of helplessness.

Past family type was also identified as an important predictor of depressive symptoms. Only 50% of the participants who formerly were part of a joint family had depressive symptoms while 69.4% of those from nuclear families had depressive symptoms in our study. A possible reason behind this finding could be that those with joint families feel more satisfied and happier to have spent time with their family members in contrast to nuclear families. Similarly, there was a significant association between presence of chronic health problems and depressive symptoms. The association between chronic medical illnesses and depressive symptoms has also been established in other studies [22, 23]. People with chronic ailments like arthritis, diabetes mellitus, heart diseases and chronic pulmonary diseases have been found to be at a higher risk of developing depression [23]. The functional limitation brought about by these chronic illnesses are considered as the most important factor leading to depressive symptoms [22].

There is also a close association between feelings of loneliness and depressive symptoms. Studies have shown that loneliness leads to serious health consequences; including depression. Those of higher age groups are reportedly found to have higher rates of loneliness due to loss of spouse, family, and social disengagement [24]. This is largely due to having to live in aged care homes after losing their family members, or partner or being separated from their family members. Loss of family members and abandonment ultimately render older adults with feelings of helplessness and loneliness.

Study characteristics like ethnicity, marital status, education and source of income were not found to have statistically significant association with depressive symptoms. In contrast, some studies have found ethnicity and education to be associated with depressive symptoms [21, 25]. A possible explanation for the variability is that these studies were community-based with participants from a diverse group of communities within the sample. There is limited literature exploring the association between marital status and source of income with depressive symptoms.

## Strengths and limitations

This study provides a fresh insight on the mental health status of older adults in aged care homes in Kathmandu Metropolitan City. To our knowledge, no previous studies have been focused in this geographical area. The study also studied socio demographic, health related and individual factors for this study. Moreover, it sheds light on neglected issue of mental health of the older adults.

The results of our study should be interpreted in light of several limitations. As the study is conducted with a cross-sectional design, causal inference cannot be established. Face to face interview approach was adopted as a majority of the respondents did not know how to read and write. As the aged care homes consist of a significant number of older adults living with disability, further research with appropriate methodology on them would be better. The outcomes of the study also cannot be generalized to any other region of the country.

## Conclusion

This study has revealed an alarming prevalence of depressive symptoms 58.8% among the older adults living in aged care homes of Kathmandu Metropolitan City. Depressive symptoms were found to be directly associated with age, sex, past family type, chronic physical health problems and feelings of loneliness. This reflects the dire need of prioritizing mental health of the older adults and calls for immediate as well as sustainable intervention measures. Timely identification and appropriately planned interventions can help reduce the burden. This could be possible with direct collaboration with health institutions and universities. Reformed policies, robust institutions, procedures and bolstering cooperation are crucial to address the problem.
